# Primary envelopment of Kaposi’s sarcoma-associated herpesvirus at the nucleoplasmic reticulum

**DOI:** 10.1128/jvi.00588-25

**Published:** 2025-10-01

**Authors:** Alexa Wilson, Neale D. Ridgway, Craig McCormick

**Affiliations:** 1 Department of Microbiology & Immunology, Dalhousie University 3688, Halifax, Nova Scotia, Canada; 2 Departments of Pediatrics and Biochemistry & Molecular Biology, Dalhousie University 3688, Halifax, Nova Scotia, Canada; Northwestern University Feinberg School of Medicine, Chicago, Illinois, USA

**Keywords:** virus, herpesvirus, KSHV, capsid, nucleus, egress, nucleoplasmic reticulum, phosphatidylcholine

## Abstract

Herpesvirus egress begins with primary envelopment of newly assembled capsids at the inner nuclear membrane (INM). Primary envelopment has been observed at the peripheral INM as well as nuclear infoldings. Nuclear infoldings from invaginations of the INM are Type-I nucleoplasmic reticulum (NR), whereas infoldings of both INM and outer nuclear membrane (ONM) are Type-II NR. Here, we report that Kaposi’s sarcoma-associated herpesvirus (KSHV) reactivation from latency and lytic cycle progression correlates with increases in both types of NR, but primary envelopment is restricted to peripheral INM and Type-I NR. These Type-I NR structures co-localized with puncta containing CTP:phosphocholine cytidylyltransferase (CCTα), the enzyme that catalyzes the rate-limiting step in phosphatidylcholine (PtdCho) synthesis that drives the *de novo* membrane biogenesis and membrane curvature required for NR expansion; CCTα recruitment may provide sufficient Type-I NR to facilitate nuclear egress. Despite the concurrent expansion of Type-II NR, primary envelopment involves a mechanism that specifically targets capsids to Type-I NR. Building upon our observation of capsids lacking envelopes in complex higher-order Type-I NR structures, we used polar lipid dyes, CLICK-labeled fluorescent viral genomes, and fluorescent KSHV capsids to track the fate of NR-associated capsids via live cell microscopy. These studies provide evidence for trafficking of NR-associated capsids toward the nuclear periphery and cytoplasm. Taken together, these findings suggest that nuclear egress occurs not only at the nuclear periphery but also at the Type-I NR.

**IMPORTANCE:**

Herpesvirus capsids assemble in the cell nucleus but are too large to exit via nuclear pores. Instead, they bud into the inner nuclear membrane to acquire a provisional lipid envelope that is shed through fusion with the outer nuclear membrane, delivering the capsid to the cytoplasm for subsequent steps in assembly and egress. These nuclear membranes are dynamic, with the ability to fold into invaginations that access the nuclear interior. Here, we demonstrate that during Kaposi's sarcoma-associated herpesvirus (KSHV) replication, nuclear membrane infolding increases, coinciding with recruitment of a host enzyme required for PtdCho synthesis at these sites. We observed accumulation of KSHV capsids at infoldings of the inner nuclear membrane and tracked the association and trafficking of fluorescent viral particles through these structures by live cell microscopy. This complements a more well-established mechanism of KSHV egress at the nuclear periphery and suggests versatility in nuclear egress mechanisms.

## INTRODUCTION

Herpesvirus capsid assembly and DNA packaging occur in a multistep process in the cell nucleus, yielding at least three distinct products known as A-, B-, and C-capsids (1, 2). All capsid types possess angular shells consisting of major capsid protein (MCP) arranged into pentons and hexons (3), small capsid protein (SCP) that caps the outer surface of hexons (4), triplex protein complexes that link adjacent capsomers (5), and a dodecameric portal protein complex (6). A-capsids are abortive, with an empty capsid shell devoid of internal scaffolding proteins or a viral genome. B-capsids are assembly intermediates or abortive capsids that contain an inner scaffold consisting of scaffold protein and scaffold protease but no viral genome (7). C-capsids are mature capsids that have ejected scaffolding proteins and replaced them with the linear viral genome. There is evidence that C-capsids gain priority access to the host cell cytoplasm for subsequent steps in assembly and egress, at least in part, through the capsid-vertex-specific component (CVSC), a multiprotein complex enriched on the penton vertices of C-capsids; however, molecular support for this mechanism remains incomplete. These newly assembled capsids are too large to exit the nucleus through nuclear pores and instead bud at the INM into the perinuclear membrane space, acquiring an envelope in the process. This event has been termed “primary envelopment.” Enveloped viruses in the perinuclear space fuse with the outer nuclear membrane (ONM), losing their envelope in the process of gaining access to the cytoplasm.

**Fig 1:**
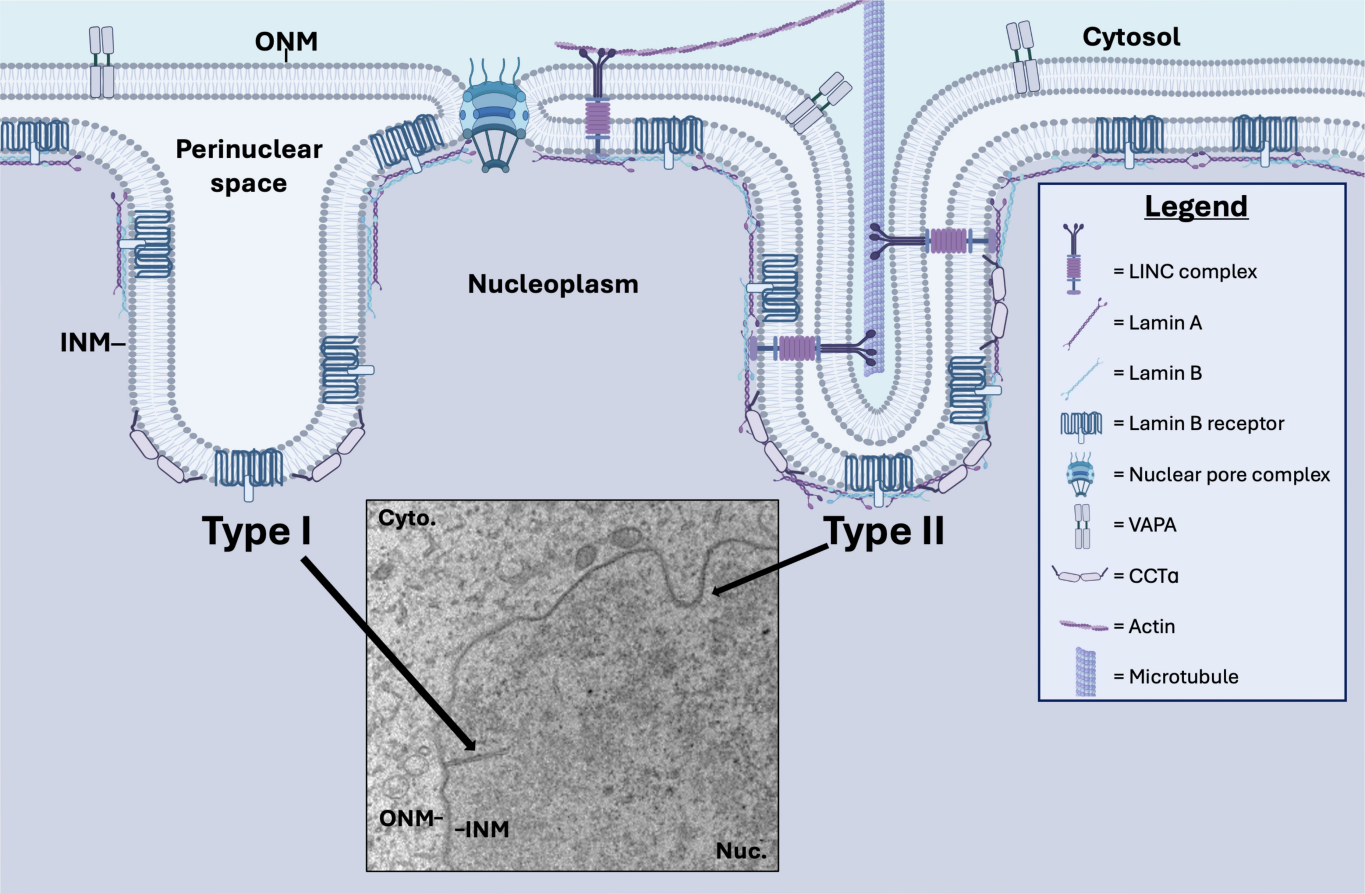
Morphology of Type-I and Type-II NR. Model depicting the ONM and INM, nucleoplasm (purple), and cytosol (blue). The inset shows an electron micrograph of Type-I NR and Type-II NR in latent iSLK cells. Invagination of the INM produces lamin-poor Type-I NR that feature INM-associated proteins, such as the lamin B receptor. Invagination of the ONM and INM together produces Type-II NR with an underlying lamina and markers including (i) lamin B receptor, (ii) vesicle-associated membrane protein-associated protein A (VAPA), which is involved in the delivery of late endosomes and nuclear transfer of extracellular vesicle-derived components to the NR, (iii) the linker of nucleoskeleton and cytoskeleton (LINC) complex, which is tethered to the cytoskeleton and regulates NM dynamics, and (iv) nuclear pore complexes. Both Type-I NR and Type-II NR feature CTP:phosphocholine cytidylyltransferase α (CCTα), an enzyme associated with the INM that catalyzes the rate-limiting step in phosphatidylcholine synthesis required for membrane biogenesis.

The generally accepted model of herpesvirus egress is primary envelopment at the peripheral INM, but there is growing evidence for an alternative mechanism involving envelopment at nuclear infoldings (NI) (8–11). NI share structural similarities with the nucleoplasmic reticulum (NR); “NI” describes any membrane infoldings that extend into the nucleoplasm, whereas “NR” refers to a subset of these structures that meet the definition of nucleoplasmic reticulum (12). Type-I NR features single-membrane branched or unbranched invaginations of INM that extend into the nucleoplasm (Fig. 1). Type-II NR are double-membrane invaginations of both inner and outer NM. As such, Type-II NR contains nuclear pores and nuclear lamina, facilitating an extended cytoplasmic interface with the nucleoplasm, whereas the Type-I NR is lamin-poor and devoid of proteins associated with the ONM. Hybrid structures incorporating both Type-I and Type-II morphology have been reported (e.g., a Type-II invagination that gives rise to multiple Type-I extensions) (12). These hybrid structures contribute to the architectural complexity of the NR.

It has long been recognized that cytomegalovirus (CMV) egress involves primary envelopment at NI. As early as 1964, intranuclear structures resembling NI were reported during murine CMV (MCMV) infection (13). In the years that followed, intranuclear compartments were documented with heightened detail during MCMV infection, and their role in primary envelopment became evident (14). Eventually, intranuclear structures were documented for human CMV (HCMV) as well (15), which were later referred to as “pseudo-inclusions” of the nucleus (16). More recent reports demonstrate that although NI account for a small fraction of nuclear membrane area during CMV infection, most capsids bud at NI rather than the peripheral INM (9). Analysis of HCMV-infected cells using focused ion beam/scanning electron microscopy (FIB/SEM) tomography revealed complex networks of tubules and spherical compartments, with the latter assuming a variety of hierarchical structures consistent with the Type-I NR (10, 12). It has been well documented that lytic herpesvirus replication causes the nucleus to increase in size (17). Villinger et al. proposed a “pushing membrane model” that considers changes in nuclear membrane size and the emergence of complex NR networks observed in HCMV-infected cells (10). In this model, disruption of the nuclear lamina is accompanied by the synthesis of new membrane, resulting in the invagination of the INM into the nucleoplasm to form complex membrane structures and providing numerous opportunity sites for HCMV budding. Similar to CMV, nuclear pseudorabies virus (PRV) capsids bud into membranous nuclear structures called “tegusomes” that resemble the NR (8). Tegusomes are sometimes connected with the NE and open into the cytoplasm, and most comprise a single membrane with occasional double-membrane structures. Inspecting TEM images of tegusomes reveals remarkable similarity to the Type-I NR and Type-II NR, as well as complex structures defined more recently by Buser et al*.* and Villanger et al*.,* which we now know to be the Type-I NR (9, 10, 18). Together, these studies provide ample evidence for herpesvirus utilization of NI for budding.

Primary envelopment of herpesvirus capsids requires the viral nuclear egress complex (NEC), which consists of a type-II transmembrane protein and a soluble phosphoprotein that assemble into a hexameric lattice structure on the INM to promote local membrane curvature required for capsid budding into the perinuclear space (19). The PRV NEC induces local INM curvature and drives the formation of vesicles embedded within the Type-I NR (20–23). Similarly, the NEC of murine gammaherpesvirus-68 (MHV-68) promotes nuclear invaginations bearing lamin A/C (24). Ectopic expression of Kaposi’s sarcoma-associated herpesvirus (KSHV) NEC proteins elicited formation of nuclear structures described as “vesicles wrapped by membranous structures in the nucleoplasm that appeared to have no connection to the nuclear membrane” (24). During herpes simplex virus type 1 (HSV-1) infection, the NEC proteins localize to the INM and induce vesicle formation (19). The HSV-1 NEC promotes the reorganization of the lamina, which acts as a structural barrier to capsids accessing the INM, causing lamin-positive protrusions in the intranuclear space that resemble the NR (25). During HSV-1 infection, the viral US3 kinase and a complex comprising the NEC, the viral protein γ_1_34.5, host scaffold protein p32, and protein kinase C, phosphorylate and destabilize the nuclear lamina (26–30). Similarly, viral serine/threonine kinases from herpes simplex virus type 2 (HSV-2) (31), Epstein-Barr virus (EBV) (32), and HCMV (33, 34), all phosphorylate and destabilize the lamina. Thus, lamina disassembly is not only a prerequisite for NR formation but also a conserved feature of herpesvirus replication.

To date, the utilization of NI for gammaherpesvirus primary envelopment remains unresolved. One study of KSHV infection demonstrated the presence of an intranuclear double-membrane structure separated from the nuclear envelope, but the authors did not comment on the nature of this invagination (35). Another study of MHV-68-infected cells reported INM invaginations that contained enveloped capsids and secondary compartments within these invaginations that contained non-enveloped capsids (11). Here, we report that KSHV reactivation from latency and lytic cycle progression correlates with increases in both Type-I NR and Type-II NR, but we only observed primary envelopment at Type-I NR and the peripheral INM. The selective utilization of Type-I NR for primary envelopment despite the concurrent expansion of Type-II NR suggests a mechanism to selectively target KSHV capsids to these membranes for budding. Over a time course of infection, we frequently observed DNA-containing KSHV C-capsids budding into nuclear infoldings contiguous with the INM and sparsely decorated with nuclear lamina, characteristic of Type-I NR. These Type-I NR structures co-localized with puncta containing CTP:phosphocholine cytidylyltransferase (CCTα), the enzyme that catalyzes the rate-limiting step in PtdCho synthesis that drives the *de novo* membrane biogenesis and membrane curvature required for NR expansion. Thus, CCTα activity may provide sufficient Type-I NR to match requirements for KSHV nuclear egress. Finally, we employed live-cell imaging approaches to track newly assembled KSHV capsids as they transited through NR structures to the cytoplasm. Together, these approaches provide a robust framework to investigate the role of the NR in KSHV nuclear egress.

## RESULTS

### Nuclear infolds increase during KSHV lytic replication

**Fig 2:**
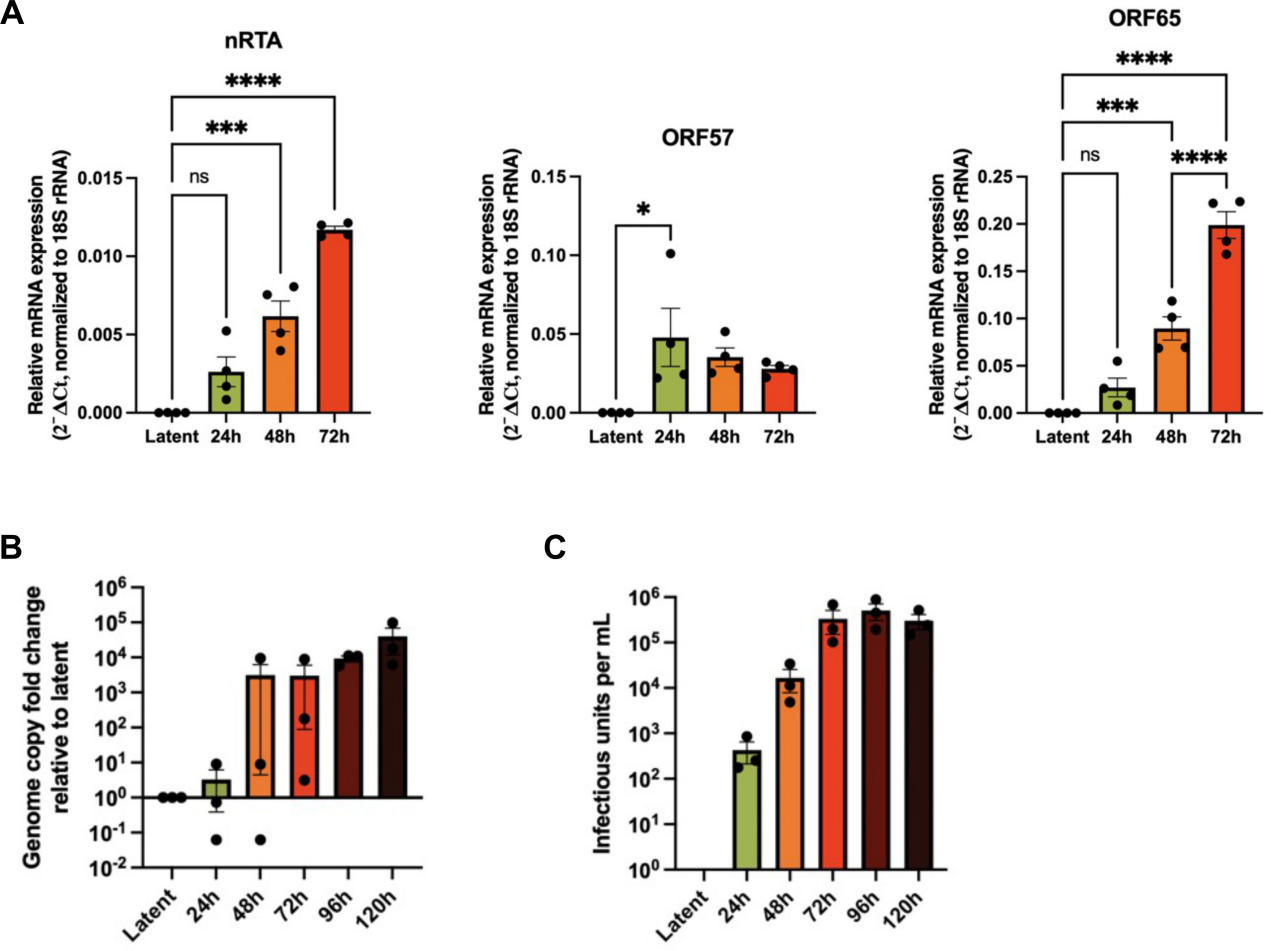
Production of infectious KSHV virions from iSLK-BAC16-GFP cells peaks at 72 hours post-reactivation. (**A**) RT-qPCR analysis of viral mRNA accumulation during lytic replication. iSLK-BAC16-GFP cells were reactivated from latency with 1 µg/mL doxycycline and 1  mM sodium butyrate. Total RNA was harvested at the indicated timepoints, and expression of the immediate early gene *native RTA* (*nRTA*) (*nRTA* is transcribed from the viral genome and can be distinguished from the *RTA* mRNA expressed in *trans*), early gene *ORF57*, and late gene *ORF65* was measured by RT-qPCR. Transcript levels were normalized to 18S rRNA using the ΔCt method (*N* = 4 biological replicates; mean ± SEM; significance determined by one-way ANOVA with Tukey’s post hoc test). *P ≤ 0.05; ***P ≤ 0.001; ****P ≤ 0.0001; ns, non-significant. (**B**) Quantification of viral genome replication. Genomic DNA was extracted from iSLK-BAC16-GFP cells at the indicated timepoints, and fold change in viral genome copies was measured by qPCR targeting *ORF26*, normalized to *β-actin* (*N* = 3 biological replicates; mean ± SEM; one-way ANOVA with Tukey’s post hoc test). (**C**) Quantification of infectious virus production. Supernatant from reactivated iSLK-BAC16-GFP cells was harvested at the indicated times and used to infect naïve 293 A cells. At 24 h post-infection, cells were fixed and stained with Hoechst, and GFP-positive cells were quantified using Imaris software (*N* = 3 biological replicates; mean ± SEM; one-way ANOVA with Tukey’s post hoc test).

NI described in previous studies of herpesvirus infection have many of the characteristics of the Type-I NR (9, 10, 12). To study NI during latent and lytic phases of KSHV infection, we used a well-established model system consisting of inducible SLK (iSLK) cells latently infected with KSHV BAC16 strain (iSLK-BAC16 cells); treatment of these cells with doxycycline (dox) and sodium butyrate stimulates ectopic expression of the viral replication and transcription activator (RTA) lytic switch protein and reactivation from latency (36). Using reverse transcription quantitative PCR (RT-qPCR), we characterized the progression of lytic replication in iSLK-BAC16 cells by measuring the accumulation of representative gene products from different stages of the lytic replication cycle, including transcripts from immediate early gene *RTA*, early gene *ORF57*, and late gene *ORF65* (Fig. 2A). iSLK-BAC16 cells have two sources of RTA: the dox-inducible *RTA* from a transgene stably integrated into the cellular genome and native *RTA* (*nRTA*) derived from the viral genome; we used oligonucleotide primers specific for *nRTA* to selectively measure *RTA* transcript produced by the viral genome. *nRTA* transcript levels increased steadily throughout the 72 hour (h) time course, demonstrating reactivation from latency in this model (Fig. 2A). RTA promotes the transcription of early genes, including *ORF57*; we observed maximal *ORF57* transcript levels at 24 h post-reactivation. As expected, the levels of the ORF65 transcript that encodes the small capsid protein peaked at 72 h post-reactivation, marking progression to late lytic replication (Fig. 2A). Viral genome replication is required to license late gene expression (37). We used a qPCR assay to demonstrate that genome replication sharply increased between 24 h and 48 h with little additional increase thereafter (Fig. 2B). The production of infectious KSHV virions was measured by collecting cell supernatants over time and titering them on naïve HEK 293 A cell monolayers and enumerating infected cells expressing the GFP reporter from the BAC16 genome. We observed that while infectious virions were produced at 24 h and 48 h post-reactivation, production peaked at 72 h post-reactivation and increased very little thereafter (Fig. 2C). Based on this finding, we selected the 72 h time point as the optimal window for analysis of KSHV nuclear egress.

**Fig 3:**
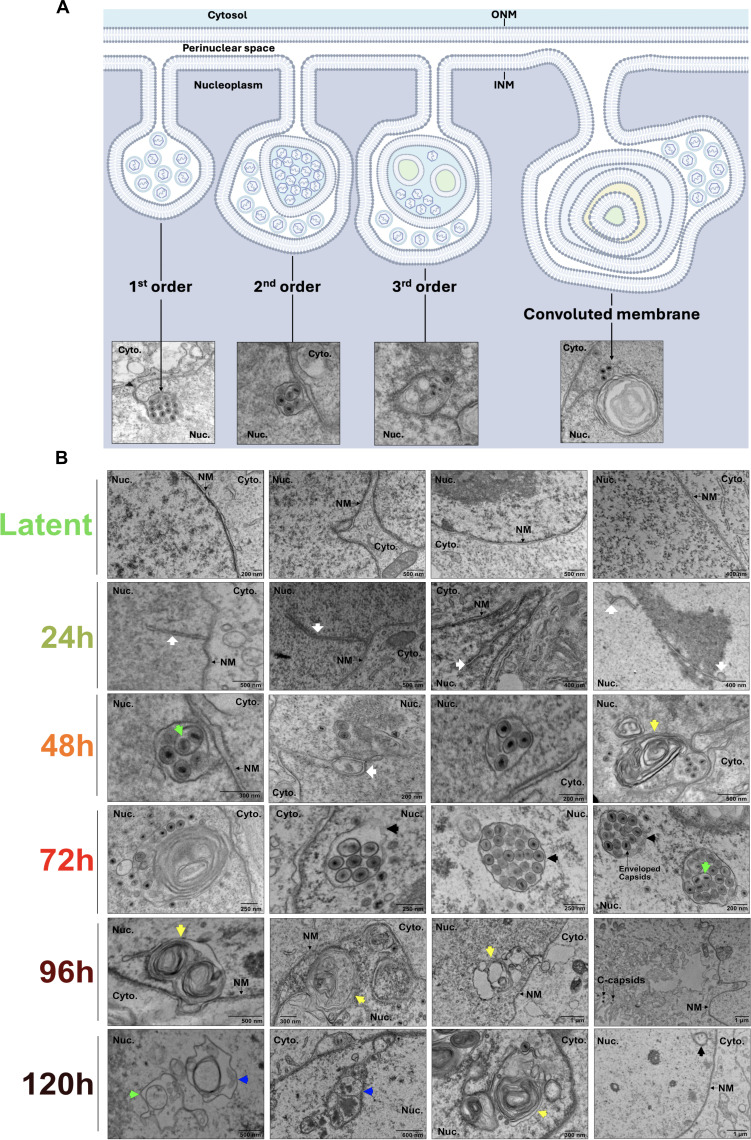
Enveloped KSHV C-capsids accumulate in diverse nuclear infolds. (**A**) NI exhibit a hierarchical order (first order = 1 infold, second order = 2 infolds, etc.). The ONM and INM are shown in relation to the cytosol (blue) and nucleoplasm (purple). First-order NI appear as single membrane tubules/vesicles that are continuous with the Type-I NR (example image reproduced from Fig. 5). Second-order NI appear as a single membrane compartment that contains one or more additional single membrane compartments (example image reproduced from Fig. 3B). Third-order NI are second-order NI that contain additional compartments (example image reproduced from Fig. 6). Convoluted membranes often appear as a second-order infolds, but the second compartment is a large, convoluted membrane whorl (example image reproduced from Fig. 5). (**B**) Transmission electron microscopy (TEM) analysis of nuclear envelope remodeling in doxycycline-inducible iSLK cells infected with KSHV BAC16. Cells were reactivated with 1 µg/mL doxycycline and 1 mM sodium butyrate and harvested at 24, 48, 72, 96, and 120 h post-reactivation. Four representative images were selected per timepoint. White arrows mark NI. Black, blue, green, and yellow arrows specifically mark first, second, third, and convoluted membrane infolds, respectively. Nucleus (Nuc), cytoplasm (Cyto), and nuclear membranes (NM) are labeled. Representative images are from *N* = 3 biological replicates. The complete set of original electron micrographs is available via the Dryad open data publishing platform at the following link: https://doi.org/10.5061/dryad.qbkh18v9.

Transmission electron microscopy (TEM) of iSLK-BAC16 cells harvested at 72 h post-reactivation revealed the presence of NI that appear as large spheroid compartments containing enveloped C-capsids (Fig. 3A). We applied a previously established hierarchical system of NI categorization (10) to our studies of KSHV infection, whereby first-order NI contain C-capsids within a single membrane compartment and second-order NI have non-enveloped C-capsid-containing periplasm-derived vesicles within a single membrane compartment. As previously reported (10), our findings suggest that the lumen of the first-order NI is continuous with the perinuclear space. The lumen of the second-order NI is reminiscent of the nucleoplasm and hypothesized to originate from invaginations of nucleoplasm into first-order NI (10). Third-order infoldings originate from separate compartments within second-order infoldings and have a lumen that has an electron density resembling that of the perinuclear space in our studies and others (10). However, the origin of these compartments remains speculative, and further confirmatory studies are required. We also describe a novel “convoluted membrane” NI, which appeared as a single membrane containing multiple encapsulated vesicles or membrane whorls.

To visualize nuclear envelope changes over the course of viral replication, iSLK-BAC16 cells were reactivated from latency and harvested for TEM at 24, 48, 72, 96, or 120 h post-reactivation (Fig. 3B). During latency, the nuclear envelope is smooth with evenly distributed lamin (Fig. 3B, first row), and only a few small NI are observed, indicating low basal NI activity, as previously reported in other human cell lines (38, 39). By 24 h post-reactivation (early lytic replication), we observed increases in NI, appearing as short tubules radiating from the nuclear periphery that are sometimes linked to spherical compartments (Fig. 3B, second row). By 48 h post-reactivation (late lytic replication), NI expand into larger spherical vacuoles connected to the INM and often form networks (Fig. 3B, third row). C-capsids enter first-order NI, acquiring a primary envelope, whereas some naked capsids are located in second-order NI; more complex third-order NI are also evident. By 72 h post-reactivation, NI increase in abundance and form large compartments containing numerous enveloped C-capsids (Fig. 3B, fourth row). At this time, NI appear as first, second, third order, and convoluted membrane morphologies. By 96 h post-reactivation, NI are largely devoid of viral capsids and have an altered elongated appearance featuring empty first- and second-order infolds (Fig. 3B, fifth row). A few residual C-capsids can be observed in the nucleus at 96 h post-reactivation, but most are found in the cytoplasm or at the cell surface. Similarly, at 120 h post-reactivation, empty NI continue to be observed, suggesting that these compartments persist long past their peak association with viral capsids (Fig. 3B, sixth row). Table 1 provides quantitative data from all electron micrographs included in this study, enumerating capsid types and their subcellular localization at different times post-reactivation, including information about their association with various types of NI.

**TABLE 1:** Quantification of capsids in the nuclei of iSLK-BAC16 cells at 48, 72, 96, and 120 h post-reactivation^*a*^

	48 h			72 h			96 h			120 h		
Capsids in the NR	65			442			37			1		
Enveloped first-order infolds	53			309			27			1		
Non-enveloped second-order infolds	12			133			10			0		
Perinuclear space	1			8			0			0		
Total capsids in nucleoplasm	352			1,492			1,334			197		
Capsid type	A	B	C	A	B	C	A	B	C	A	B	C
Nucleoplasm	75 21%	63 18%	214 61%	139 9%	414 28%	939 63%	203 15%	861 65%	270 20%	11 6%	136 69%	50 25%
Total C-capsids in the cytoplasm	24			218			286			50		

^*a*^ Total capsids were counted for all TEM images included in this study. Capsids were categorized as follows. (i) NI-associated capsids, further subdivided into capsids located in first-order infolds and capsids located in second-order infolds, and (ii) capsids associated with the periplasm. (iii) Total nuclear capsid counts include every capsid found in the nucleus. Total nuclear capsids have been further divided into A-, B-, and C-capsid subtypes. (iv) Total cytoplasmic capsids were quantified from 36 cells, including any capsids in the process of actively budding from the cell membrane.

**Fig 4:**
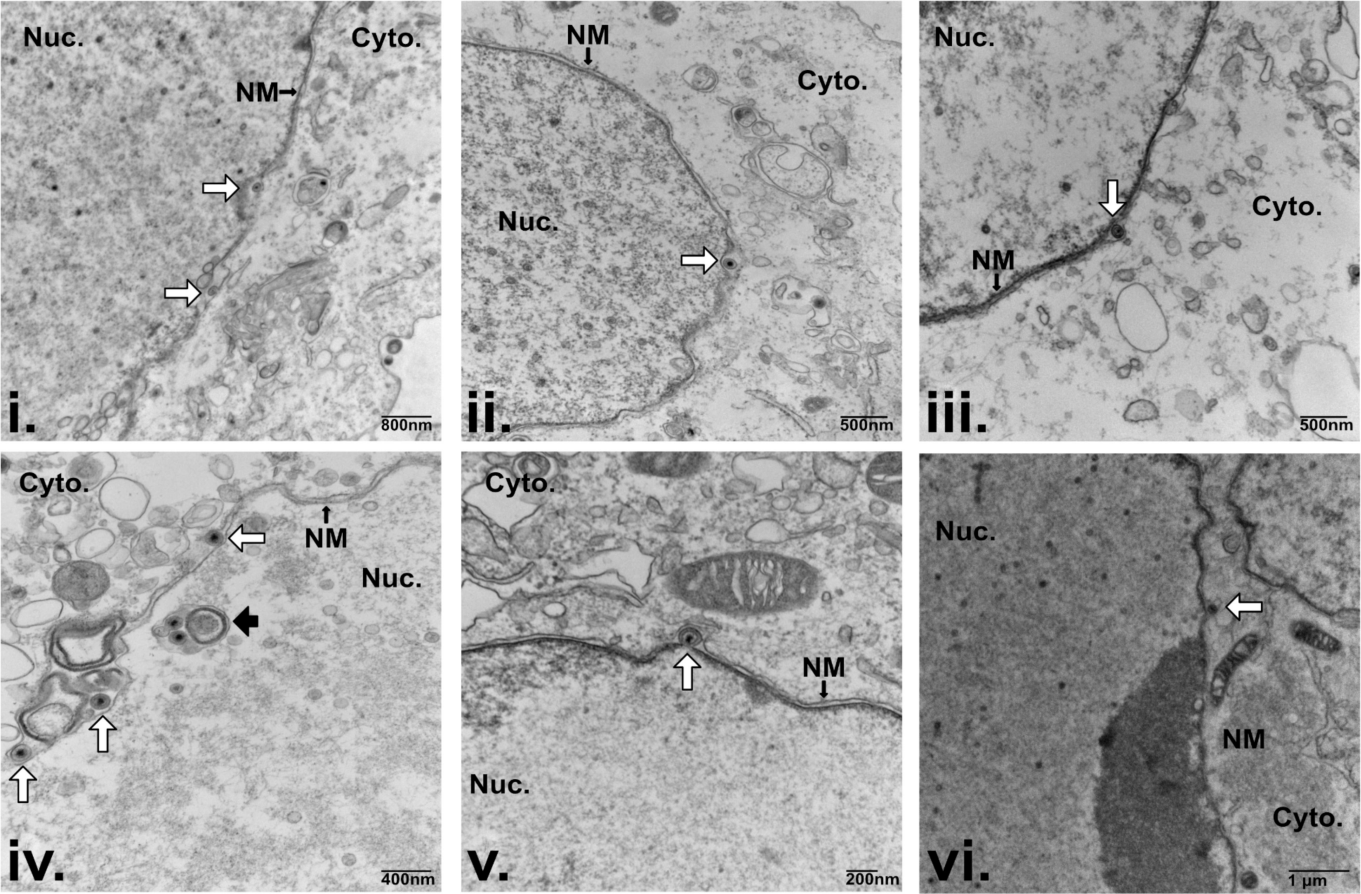
KSHV C-capsids can undergo primary envelopment at nuclear infoldings and the peripheral inner nuclear membrane concurrently. Electron micrographs of iSLK-BAC16 cells reactivated from latency by the addition of 1 µg/mL doxycycline and 1 mM sodium butyrate and harvested at 72 hours post-reactivation. Nine individual primary envelopment events at the peripheral INM are shown. Image “v.” shows a nuclear infold containing enveloped C-capsids (black arrow) with three canonical peripheral budding events nearby, marked by white arrows.

During peak activity at 72 h post-reactivation, NI-containing enveloped KSHV C-capsids were often observed within single membrane compartments with first-order NI morphology (Fig. 3B, black arrows). However, C-capsids were also observed budding into single membrane structures surrounding a multi-membrane whorl (Fig. 3B, yellow arrows). In addition, a subset of NI appeared to have a second-order infold structure, and vesicles or tubules were seen inside a compartment akin to first-order NI (Fig. 3B, blue arrows). Finally, although we captured significantly more capsids budding into NI, we still observed “classical” budding at the peripheral NM at 72 h post-reactivation (Fig. 4, white arrows). For example, we observed a cell that contained three classical budding events at the peripheral NM, alongside a complex NI that contained multiple enveloped C-capsids within a first-order NI (Fig. 4 panel iv, black arrow). Taken together, these observations indicate that KSHV C-capsids are associated with diverse NI structures, and envelopment at the peripheral NM and envelopment at NI can take place concurrently in an infected cell.

### KSHV buds into Type-I nucleoplasmic reticulum

**Fig 5:**
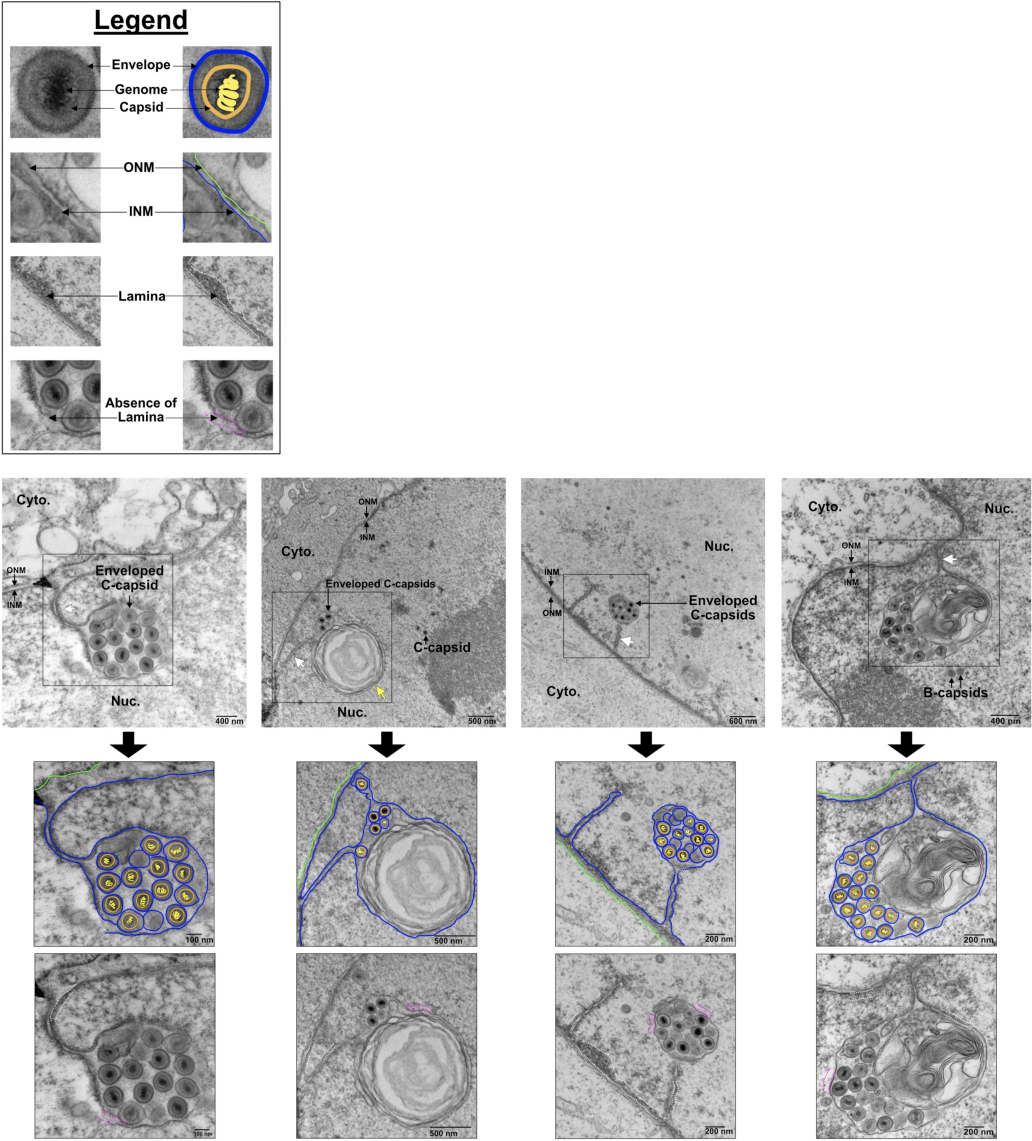
Primary envelopment of KSHV C-capsids at Type-I nucleoplasmic reticulum. Electron micrographs of iSLK-BAC16 cells reactivated from latency by the addition of 1 µg/mL doxycycline and 1 mM sodium butyrate and harvested at 72 h post-reactivation. An electron-dense matrix that resembles nuclear lamina is marked by white dashed lines across different NR regions. White arrows mark tubules connecting intranuclear compartments in the INM. The yellow arrow points to a convoluted membrane. Duplicate images are colorized to aid interpretation: INM (blue), ONM (green), enveloped C-capsids (blue), capsids (orange), and nucleoproteins (yellow). Discontinuities in the INM are indicated by blue dotted lines. A legend is provided to help the reader inspect TEM images and identify key features, including envelope, genome, capsid, ONM, INM, lamina, and absence of lamina. The images associated with the legend were reproduced from our collection of electron micrographs and include a capsid presented in Fig. 8. The complete set of original electron micrographs is available via the Dryad open data publishing platform at the following link: https://doi.org/10.5061/dryad.qbkh18v9.

We observed accumulation of enveloped KSHV C-capsids in NI that resemble the Type-I NR. However, definitive identification of these compartments as Type-I NR requires evidence of their direct connection to the INM. At 72 h post-reactivation, we observed enveloped KSHV C-capsids within NI that were clearly connected to the INM, but not the ONM, by a tubule (Fig. 5, white arrows; blue lines trace continuity with the INM). An electron-dense layer resembling the nuclear lamina extends along the neck of the Type-I NR but appears largely absent around spheroid compartments containing enveloped C-capsids (Fig. 5, white dashed lines). In some cases, this dense layer is challenging to distinguish from peripheral heterochromatin, and we therefore acknowledge the possibility that, in certain images, the observed density may include contributions from both the lamina and adjacent chromatin. Our observations are consistent with established literature describing Type-I NR, which typically feature a small amount of nuclear lamina limited to regions adjacent to the peripheral INM (12, 40). These observations are also consistent with the lamin-poor Type-I NR observed during MCMV infection (9). By contrast, even though Type-II NR also expands during KSHV lytic replication, and we observed C-capsids adjacent to Type-II NR, we have never observed budding events at these structures.

**Fig 6:**
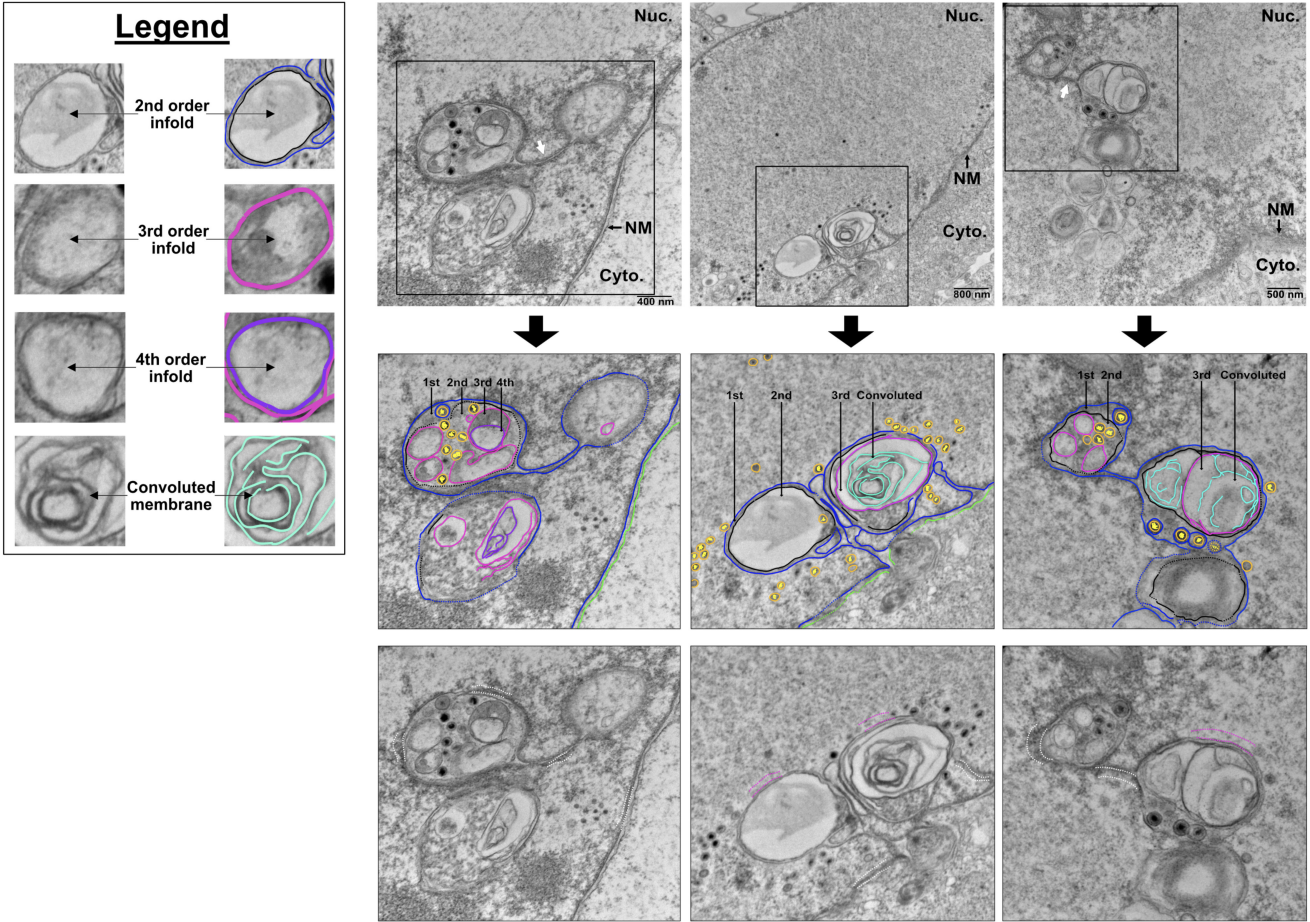
Type-I NR forms hierarchical membrane networks comprised of interconnected spherical compartments and tubules. Electron micrographs of iSLK-BAC16 cells reactivated with 1 µg/mL doxycycline and 1  mM sodium butyrate and harvested at 72 h post-reactivation reveal complex Type-I NR morphologies. Compartmental Organization includes first-, second-, third-, and fourth-order infoldings, as well as highly convoluted membrane structures. These nested compartments are interconnected via tubules, suggesting a dynamic, multi-layered membrane architecture. Enveloped C-capsids are visible within first-order infolds.

During HCMV infection, Type-I NR is a complex consisting of a hierarchy of compartments interconnected by tubules (10). Similarly, we documented numerous instances where spherical compartments containing enveloped C-capsids were connected to other spherical compartments by a tube-shaped “neck,” resulting in the formation of a network (Fig. 6). We frequently observed C-capsids budding into 1st order NI surrounding, and/or sometimes appearing to be contiguous with, convoluted membranes, but C-capsid access to the convoluted membrane interior was exceedingly rare, suggesting a potential barrier in capsid access to convoluted membrane structures.

Taken together, our observations indicate that NI associated with KSHV C-capsids during infection are lamin-poor and continuous with the INM, meeting the definition of the Type-I NR (12). Similar to HCMV infection (10), the Type-I NR is remodeled into membranous networks during KSHV infection, characterized by multiple spherical compartments connected by tubule necks to the INM.

### Nucleoplasmic reticulum expansion in KSHV-infected cells correlates with recruitment of CTP:phosphocholine cytidylyltransferase and increased intranuclear VAPA

We next conducted immunofluorescence microscopy studies on KSHV-infected cells to determine the subcellular localization of key proteins involved in NR expansion, lamin A/C, VAPA, and CCTα (41). VAPA engages oxysterol-binding protein–related protein 3 and Rab7 to form the VOR complex responsible for the transfer of extracellular vesicle (EV)-derived components from endosomes into the NR. This mechanism delivers cargo from internalized EVs to specific regions in the nucleoplasm, often near the nucleolus. CCTα is activated by insertion of a lipid-sensing amphipathic helix into the INM, where it catalyzes the rate-limiting step for PtdCho synthesis required for *de novo* membrane biogenesis (42). CCTα also aids in NR expansion by promoting membrane curvature in collaboration with a lamin A/B1 scaffold (Fig. 1). Since the ER is continuous with the ONM, VAPA marks Type-II NR, whereas CCTα is associated with both Type-I and II NR.

**Fig 7:**
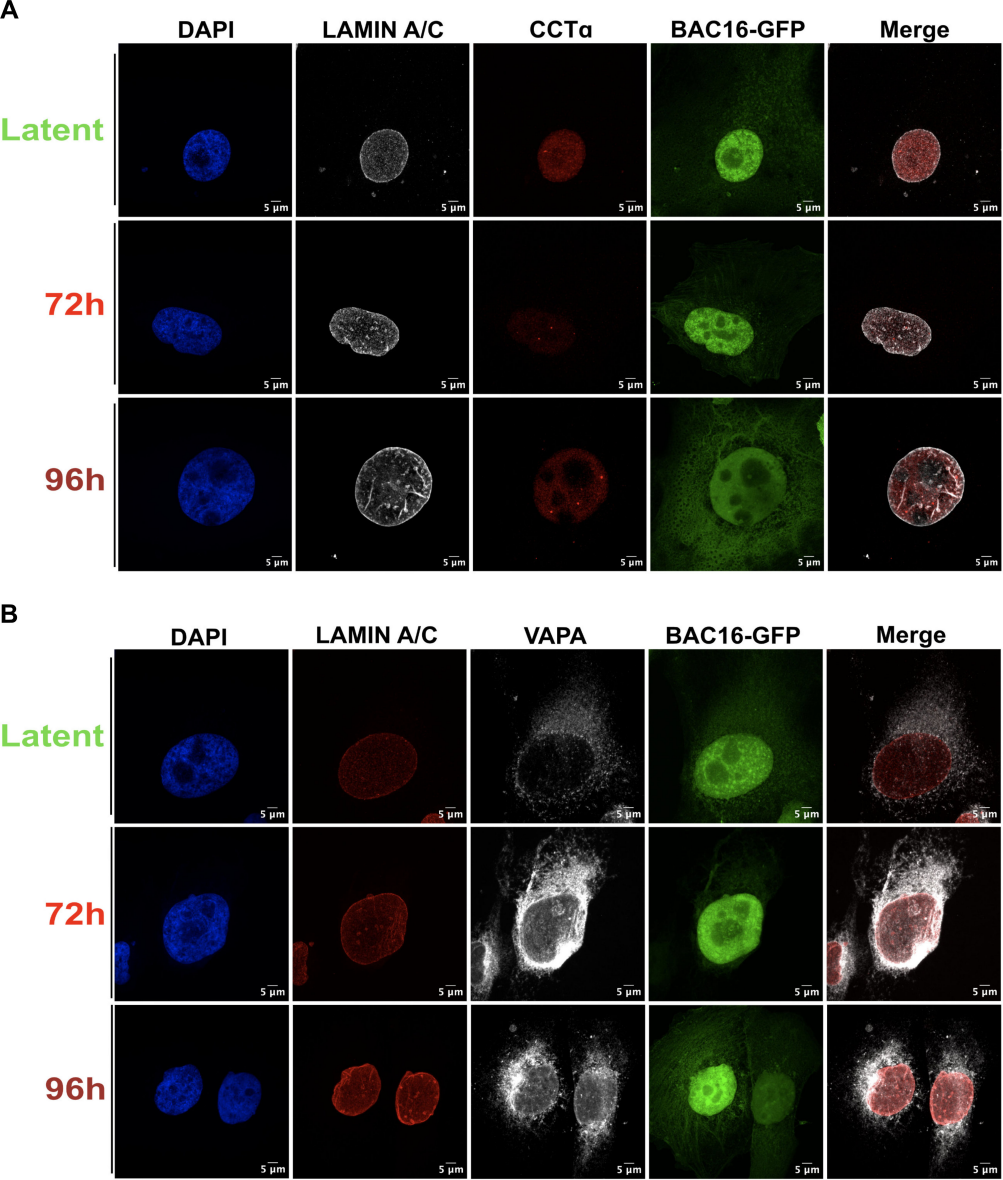
Nucleoplasmic reticulum expansion in KSHV-infected cells correlates with the emergence of CTP:phosphocholine cytidylyltransferase puncta and increased intranuclear VAPA. (**A and B**) iSLK-BAC16 cells cultured on coverslips were reactivated from latency by the addition of 1 µg/mL doxycycline and 1  mM sodium butyrate and harvested at 72 and 96 h post-reactivation. Cells were fixed and immunostained for lamin A/C and CCTα (**A**) or lamin A/C and VAPA (**B**). All images were acquired using a confocal laser scanning microscope and are presented as maximum intensity projections from one of three independent experiments.

In latently infected cells, lamin A/C is uniformly distributed across the INM and intensified at the nuclear envelope edges (Fig. 7A and B). By 72 h post-reactivation, lamin A/C projections radiate from the INM, forming spherical compartments, consistent with NR expansion reported in previous studies of the NR (43, 44) and lamin reorganization during HCMV, EBV, HSV-1, and HSV-2 infection (31, 32, 45, 46). By 96 h, lamin A/C-positive tubules emerged from the INM, exhibiting a branched, cylindrical morphology and linking multiple NR compartments. One limitation of this lamin A/C immunostaining procedure is that it does not detect lamin-poor NR compartments that were readily detected by TEM; instead, these immunostaining images show lamin A/C-positive tubules lacking a terminal compartment, which is likely undetectable due to lamin deficiency.

CCTα staining in latently KSHV-infected cells was uniformly distributed in the nucleoplasm, with only occasional puncta, suggesting that CCTα primarily remains in its inactive state (Fig. 7A). By 72 and 96 h post-lytic reactivation, CCTα puncta were prominent, indicating activation at membranes within the nucleoplasm. These CCTα puncta partially overlapped with lamin A/C-positive structures, suggesting that CCTα localizes to compartments with and without lamin A/C. VAPA staining in latent cells was primarily localized to the peripheral ER, with dim puncta scattered throughout the nucleus (Fig. 7B). At 72 h post-reactivation, VAPA-positive veins and hollow loops appear near the nuclear periphery, characteristic of Type-II NR. By 96 h, bright VAPA-positive puncta and tubules extend through the nucleus, displaying partial co-localization with lamin A/C, confirming our previous observations of increased Type-I and Type-II NR in these cells by TEM.

### Selective budding of C-capsids into Type-I nucleoplasmic reticulum

**Fig 8:**
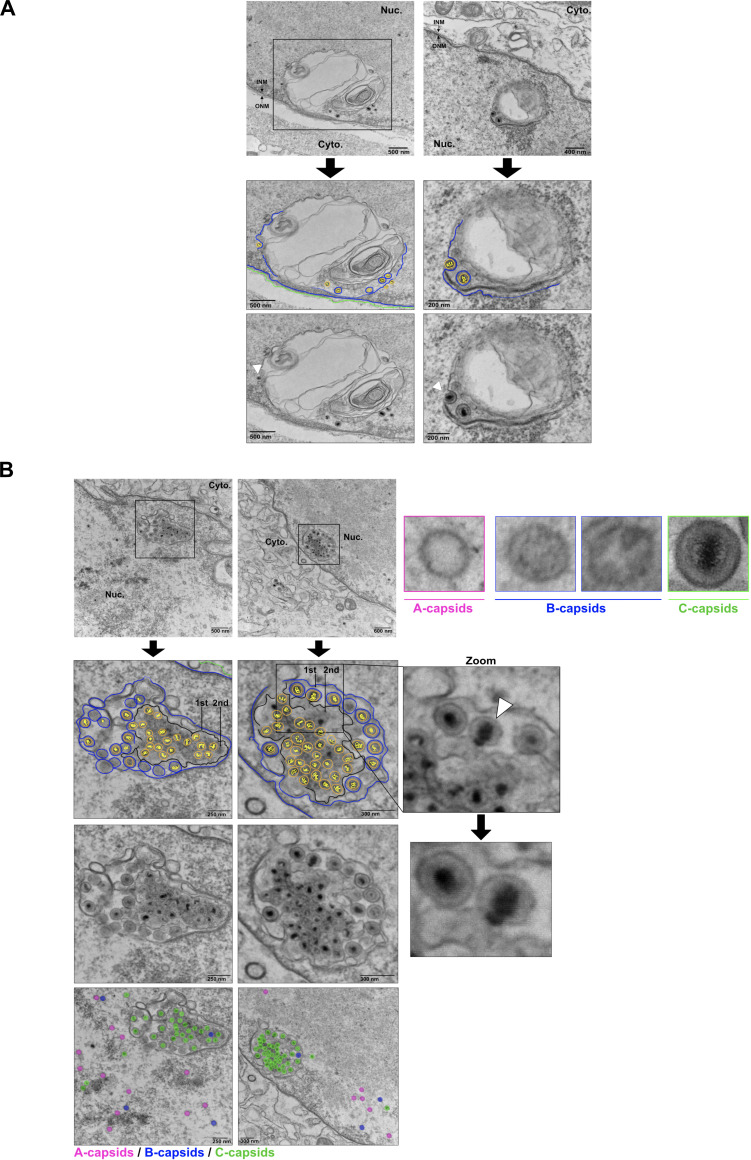
Selective budding and fusion of C-capsids into Type-I nucleoplasmic reticulum. (**A**) iSLK-BAC16 cells were reactivated from latency by the addition of 1 µg/mL doxycycline and 1 mM sodium butyrate. Cells were harvested 72 h post-reactivation and processed for transmission electron microscopy (TEM). White tailless arrows show C-capsids budding into the Type-I NR. (**B**) Unenveloped C-capsids accumulate within the second-order NI. iSLK-BAC16 cells were harvested for TEM as described for panel **A**. Zoomed-in images show a C-capsid transitioning between second-order and first-order nucleoplasmic reticulum compartments, highlighted by a white tailless arrow. First- and second-order compartments are labeled as such. A (pink), B (blue), and C (green) capsids are colorized, and representative images of each capsid type are shown on the right-hand side.

Our frequent observation of KSHV C-capsids near and within Type-I NR motivated further analysis of selective capsid recruitment and budding at these structures. Indeed, we captured events of capsid budding into Type-I NR where the surrounding membrane appeared to conform tightly around this capsid, consistent with membrane curvature changes associated with capsid envelopment (Fig. 8A). Furthermore, in nuclei containing an assortment of capsid types, we observed that C-capsids were associated with Type-I NR structures, whereas B-capsids and A-capsids were largely restricted to the nucleoplasm (Fig. 8B). We also observed complex Type-I NR structures bearing enveloped C-capsids, as well as naked C-capsids in the interior of second-order Type-I NR. These observations suggest that C-capsids can accumulate at invaginations of the nucleoplasm into the Type-I NR. Capsids from all TEM images in this study were counted and their abundance in different regions of the infected cell was recorded as follows: (i) capsids in the NR (further divided into capsids present in first-order infolds and second-order infolds), (ii) capsids in the perinuclear space, (iii) total capsids in the nucleus (including all capsids associated with the NR and peripheral NE, and (iv) total capsids in the cytoplasm (Table 1). At 48 h post-reactivation, 18% of nuclear capsids were associated with the NR compartment, increasing to a peak of 30% by 72 h post-reactivation. Most NR-associated capsids at all timepoints were localized to 1st order infolds, suggesting preferential capsid accumulation at these structures. By 96 h post-reactivation, the proportion of NR-associated capsids declined to 3% and most C-capsids were found in the cytoplasm, leaving behind a greater proportion of nuclear A- and B-capsids. These findings provide evidence for a primary envelopment pathway that specifically selects for C-capsids, where the hierarchy of Type-I NR architecture plays a role in capsid transit. Importantly, the cells analyzed were enriched for prominent NR compartments and therefore do not represent their frequency across the total lytic population.

### Dynamic labeling of viral genomes using CLICK chemistry enables capsid tracking at the nucleoplasmic reticulum

**Fig 9:**
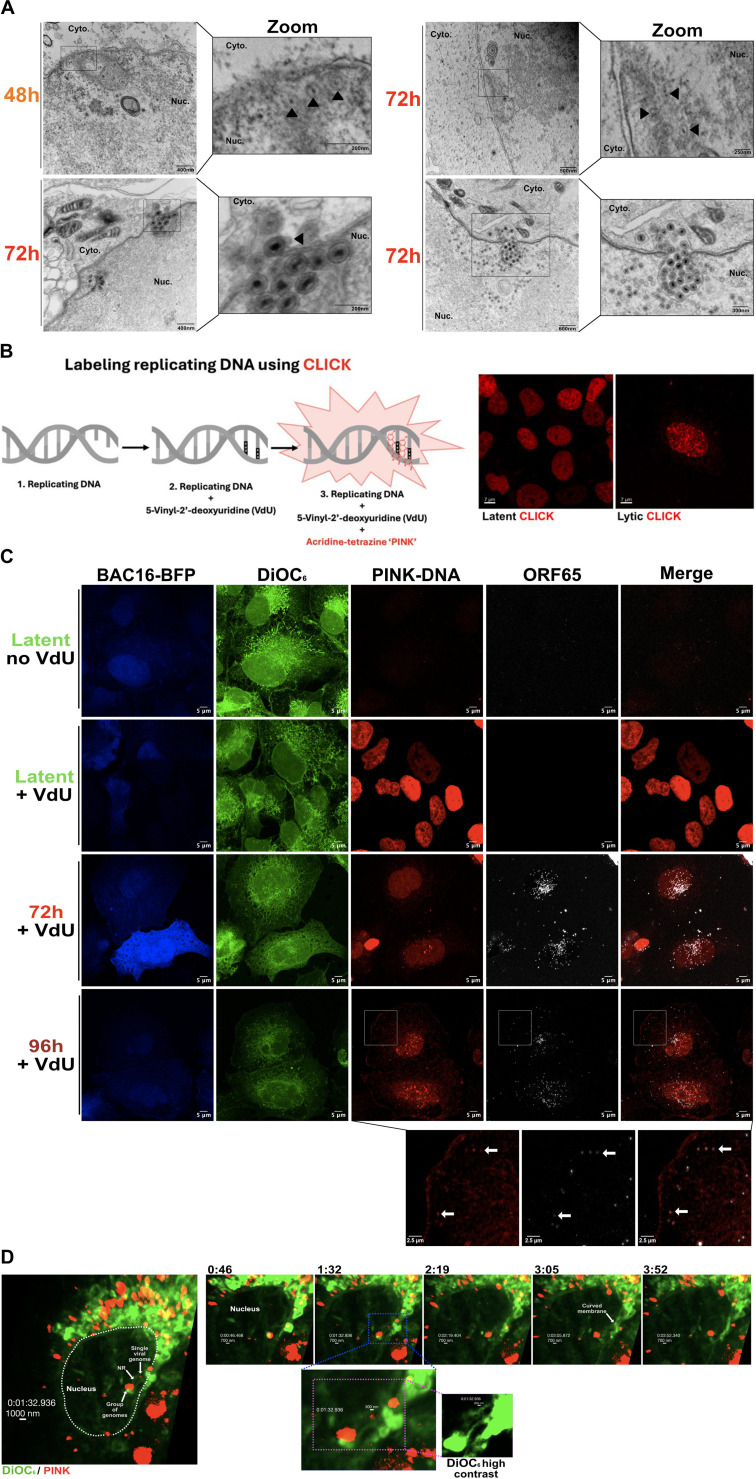
Dynamic labeling of viral genomes using CLICK chemistry enables capsid tracking at the nucleoplasmic reticulum. (**A**) iSLK-BAC16 cells were reactivated from latency by the addition of 1  µg/mL doxycycline and 1 mM sodium butyrate for the indicated timepoints and analyzed by TEM. Capsids visualized in Type-I NR necks and found invading the cytoplasm are indicated by black tailless arrows in enlarged images on the right-hand side. (**B**) Diagram of CLICK chemistry. A synthetic nucleotide analog (VdU) is incorporated into newly replicating DNA. The probe called photo-inducible nucleotide KLICK (PINK) is added to live cells. PINK intercalates into DNA and forms a covalent bond with VdU, resulting in the dequenching of PINK and fluorescence emission. Latent cells are readily labeled by PINK due to host genome replication (shown in red). In lytic cells, host genome replication is arrested, and less PINK labeling occurs, instead revealing PINK-positive puncta marking newly synthesized viral genomes. (**C**) CLICK-labeled genomes associate with the ORF65 capsid protein. iSLK-BAC16 cells cultured on coverslips were reactivated from latency by the addition of 1 µg/mL doxycycline and 1 mM sodium butyrate. Cells were harvested at 72 h and 96 h post-reactivation. Coverslips were fixed and immunostained for ORF65 (white), incubated with the lipid stain DiOC_6_ (green), and CLICK chemistry using PINK was applied (red) (*N* = 2). (**D**) Live-cell imaging of CLICK-labeled iSLK-BAC16 cells reveals exit from the nucleoplasmic reticulum. iSLK-BAC16 cells were seeded in chamber well slides and reactivated from latency by the addition of 1 µg/mL doxycycline and 1 mM sodium butyrate. To label newly synthesized DNA, cells were pulsed with VdU at 40 hours post-reactivation. At 44 hours post-reactivation, cells were pulsed with PINK (red) for 4 hours followed by three washes with warm media. At 72 h post-reactivation, cells were stained with DiOC_6_ (green), which marks the nuclear membrane and other cellular membranes containing polar lipids. Live images were captured as Z-stacks (seven slices) under 100× oil immersion at 46 second intervals (Zeiss Cell Observer Spinning-disk Confocal) (*N* = 2). White dashed lines mark the nuclear envelope at 0:00 minutes. A blue-sashed box marks an inset showing areas of potential viral genome release from the NR. The inset highlighted by a pink dashed box provides increased detail as a high-contrast image of the green DiOC_6_ channel that highlights NR structure.

Despite evidence that KSHV C-capsids can undergo primary envelopment and perhaps de-envelopment at Type-I NR and accumulate in the cytoplasm over time, it remains difficult to determine the fate of capsids by inspecting static electron micrographs. Nevertheless, we did observe capsids in the narrow “neck” of Type-I NR structures and congregating in the space between the ONM and expanded INM (Fig. 9A, black arrows). We reasoned that labeling of viral genomes prior to their loading into nascent capsids could enable tracking of capsid nuclear egress. To monitor capsid trafficking in NR compartments, we conducted live-cell imaging experiments using the green fluorescent dye 3,3′-Dihexyloxacarbocyanine Iodide (DiOC_6_) to label polar lipids, including NR, and inverse electron demand Diels-Alder (IEDDA) CLICK chemistry to label viral genomes (47). We pulsed labeled newly synthesized viral DNA with the synthetic nucleotide 5-vinyl-2'-deoxyuridine (VdU), followed by treatment with acridine tetrazine (PINK) (48); a similar approach was recently used to study replication compartments during adenovirus and HSV-1 infection (49). iSLK-BAC16 cells stably expressing blue fluorescent protein (BFP) were seeded on coverslips, reactivated from latency, and pulsed with VdU at 40 h post-reactivation. Cells were subsequently fixed, incubated with PINK, and processed for immunofluorescence imaging at 72 h or 96 h post-reactivation. In latently infected cells, nuclear DNA labeling was uniformly distributed, consistent with VdU incorporation into viral DNA as well as host DNA during S-phase. Because KSHV lytic replication triggers host cell cycle arrest (50), these cells exhibit reduced DNA labeling overall and the emergence of nuclear puncta representing newly synthesized viral DNA (Fig. 9B). These red fluorescent puncta co-localized with KSHV ORF65 (small capsid protein), confirming their identity as viral genomes that were subsequently packaged into capsids (Fig. 9C). At 72 h post-reactivation, puncta containing viral DNA and ORF65 were often clustered near DiOC_6_-labeled invaginations, confirming capsid association with the NR as we observed previously by electron microscopy. By 96 h post-reactivation, these capsid puncta largely accumulated in the cytosol, suggesting that newly assembled capsids containing labeled viral genomes were competent for nuclear egress.

We next used live-cell microscopy to track the fate of NR-associated CLICK-labeled viral genomes in real time. iSLK-BAC16 cells were reactivated from latency and pulsed with VdU at 40 h post-reactivation, followed by a PINK pulse from 44 to 48 h post-reactivation. DiOC_6_ staining allowed us to visualize the expanded NR in live cells, highlighting brightly stained compartments connected to the INM by a hollow “neck” (Fig. 9D). In the cell nucleus, DiOC_6_-positive NR co-localized with large red fluorescent clusters of newly synthesized DNA (Fig. 9D, Movies S1 and S2). In real time, we observed smaller DNA foci appear to emerge from the densely packed compartment and transit an NR neck to the peripheral NE (Fig. 9D, 1: 32 and 2:19 minutes). In the next frames, we observed the positive curvature of the NE and arrival of viral DNA puncta in the cytoplasm (Fig. 9D, 3:05 and 3:52 minutes). Thus, these images show that capsid transit from large NR compartments to the cytoplasm can occur in a few minutes. Increasing the contrast of the DiOC_6_-stained NR neck revealed intricate spherical patterns along the NR neck, resembling primary envelopes in both size (~300 nm) and shape. Based on this data, we suggest that capsids with labeled viral genomes can transit from NR compartments to the cytoplasm.

### Fluorescent KSHV capsid transit through nucleoplasmic reticulum

**Fig 10:**
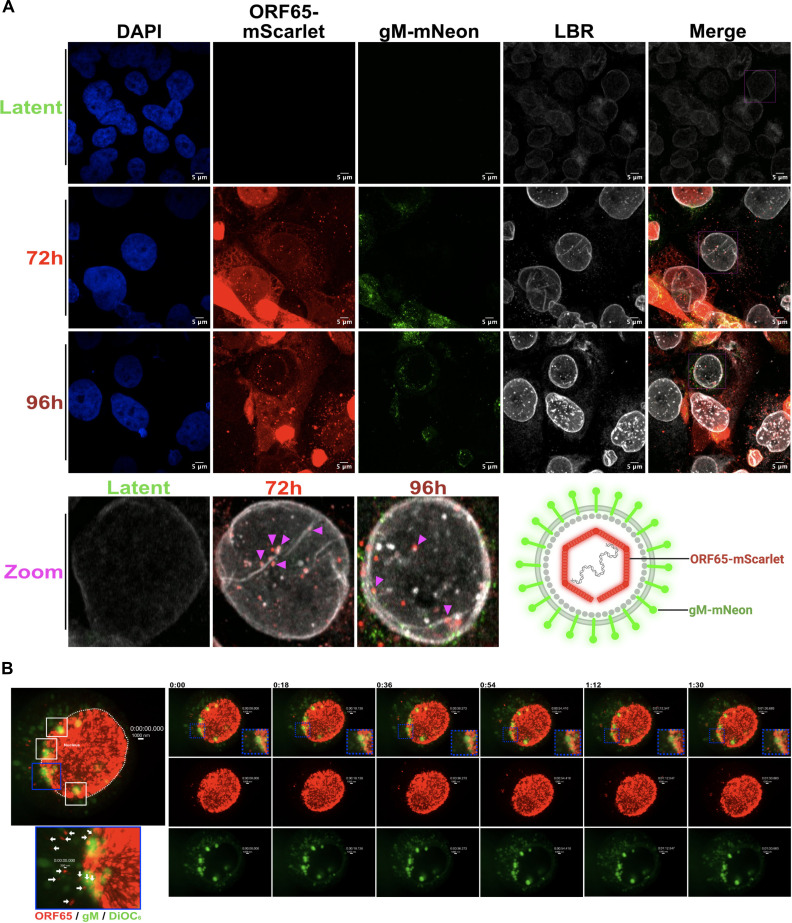
Fluorescent KSHV capsids transit through the nucleoplasmic reticulum. (**A**) Fluorescent KSHV capsids are observed at the NR. Diagram of dual-labeled KSHV virions expressing fluorescent capsid and envelope markers (bottom right cartoon). ORF65-mScarlet fusion protein enables tracing of KSHV capsids, with gM-mNeonGreen marking locations of viral envelope glycoproteins, which enables real-time tracking of capsid and envelope dynamics during infection. iSLK-ORF65-mScarlet/gM-mNeon cells were seeded on coverslips and reactivated from latency by the addition of 1 µg/mL doxycycline and 1  mM sodium butyrate. Cells were harvested at 72- or 96 hours post-reactivation and immunostained with anti-lamin-B receptor antibody to mark the NR (white) and stained with DAPI to mark nuclei (blue). Magenta boxes highlight insets shown in zoomed-in images below, enabling capsid co-localization with LBR compartments to be seen in detail at 72 h and 96 h timepoints. (*N* = 2) (**B**) Live-cell imaging shows ORF65-mScarlet-positive puncta closely associated with the nucleoplasmic reticulum (bright green circles) and appearing to shuttle from the NR into the cytoplasm. iSLK-ORF65_mScarlet–gM_mNeon cells were reactivated and stained with DiOC_6_ and imaged live using 100× oil immersion (6 Z-stacks slices taken over 18 second intervals, Zeiss Cell Observer Spinning-disk Confocal microscope, *N* = 2). Time is shown as minutes:seconds. Although both gM and DiOC₆ fluoresce in the green spectrum, DiOC₆ was used to visualize the NR during live-cell imaging. As shown in panel **A**, some perinuclear gM-mNeon signal can be observed, but it is far less intense than DiOC6-labeled NR compartments. White arrows indicate ORF65-positive puncta. White dashed lines outline the nuclear envelope at 0:00. Blue insets highlight where puncta are concentrated in and released from regions of the nucleus, where NR structures are prominent.

To further investigate the role of the NR in KSHV nuclear egress, we performed live-cell imaging using a dual-color reporter KSHV (51). This BAC16-ORF65_mScarlet-gM_mNeon virus (hereafter RG-BAC16) encodes fluorescent capsid (ORF65_mScarlet) and envelope (gM_mNeon) proteins, enabling real-time tracking of capsid assembly and nuclear egress (Fig. 10A, bottom right cartoon). At different times post-reactivation, iSLK cells infected with RG-BAC16 were stained with anti-lamin B receptor (LBR) antibody to mark both Type-I NR and Type-II NR structures (Fig. 10A). At 72 h post-reactivation, bright ORF65_mScarlet puncta appear in the nucleus, alongside more diffuse cytoplasmic staining, likely reflecting newly synthesized ORF65_mScarlet that has yet to be imported to the nucleus. ORF65_mScarlet puncta were often observed in association with LBR-positive NR that appeared as spherical compartments connected to the NE by hollow “necks.” By 96 h post-reactivation, cytoplasmic ORF65_mScarlet puncta increased and could be seen in proximity to gM_mNeon, although some ORF65_mScarlet signal remained associated with the NR and peripheral nuclear membrane. These images suggest that ORF65-mScarlet reporter protein can be used to monitor trafficking of newly assembled KSHV capsids.

To visualize trafficking of ORF65_mScarlet capsids in real time, iSLK cells infected with RG-BAC16 were seeded in chamber well slides and reactivated from latency by addition of 1 µg/mL doxycycline and 1 mM sodium butyrate. At 72 h post-reactivation, cells were stained with DiOC_6_ to label NR structures, and Z-stack images were taken on a spinning disc microscope at 18 second intervals (Fig. 10B). Distinct DiOC_6_-positive intranuclear compartments were observed around the nuclear periphery, showcasing the expanded NR, in association with ORF65_mScarlet-positive puncta (Fig. 10B, blue boxes; Movies S3 and S4). These NR-associated ORF65_mScarlet puncta moved toward the nuclear periphery over time (Fig. 10B, time 0:00 and 0:18 minutes, blue box) and accessed the cytoplasm (Fig. 10, 0:36 minutes, blue box). Even though precise mechanisms of KSHV nuclear egress via NR structures remain obscure, these movies and static images support the idea that NR-associated capsids can transit to the cytoplasm.

## DISCUSSION

During herpesvirus primary envelopment, capsids bud into INM with the help of a two-component viral NEC, briefly acquiring an envelope that is shed via fusion with the ONM. Upon gaining access to the cytoplasm, these capsids bud into the *trans* Golgi network and travel to the cell surface in secretory vesicles that fuse with the plasma membrane to release progeny herpesviruses into the extracellular environment. This primary envelopment event at the INM has been observed at the nuclear periphery, but also at NI that provide a tantalizing route for acquiring newly assembled capsids from viral replication compartments. However, mechanisms governing herpesvirus access to NI remain poorly characterized. Here, we report that both Type-I and Type-II NR increase during KSHV lytic replication, correlating with the recruitment of CCTα to these membranes, which drives membrane proliferation via PtdCho synthesis. There is emerging evidence that herpesvirus nuclear egress operates with a quality-control mechanism wherein intact DNA-containing C-capsids are selectively exported, leaving behind immature or defective capsids. Our study reveals another aspect of selectivity in KSHV nuclear egress; even though both Type-I NR and Type-II NR increase during KSHV lytic replication, we only observe budding into Type-I NR. This supports a mechanism of KSHV primary envelopment that complements the canonical model, whereby lamin-poor Type-I NR membranes that probe the nuclear interior provide a site for primary envelopment of newly synthesized KSHV capsids.

Inspecting a large collection of electron micrographs allowed us to observe KSHV budding at the Type-I NR and the peripheral INM in the same nucleus, suggesting that both mechanisms operate concurrently. These observations are consistent with previous studies of human betaherpesviruses and murine gammaherpesviruses and suggest versatility in herpesvirus nuclear egress pathways. However, we do not yet understand the factors that influence which route of primary envelopment a newly assembled KSHV capsid pursues, or their relative rates of usage. Furthermore, the factors that limit capsid budding into Type-II NR remain obscure. These undetermined mechanisms represent attractive targets for future research.

In addition to broadly promoting NR proliferation, KSHV lytic replication increased the formation of complex higher-order Type-I NR structures. We observed a striking accumulation of naked C-capsids in second-order infoldings of Type-I NR, suggesting a role for these complex structures in KSHV nuclear egress. However, the mechanism of capsid recruitment to second-order infoldings remains unknown. We also frequently observed greatly expanded Type-I NR-derived convoluted membranes, which may be the product of runaway INM expansion that causes the membrane to fold into a whorl. We speculate that CCTα drives INM expansion and convoluted membrane formation through its ability to dimerize in *trans*, linking and stabilizing opposing membranes while catalyzing the rate-limiting step in PtdCho synthesis (52). In this way, CCTα could promote unchecked INM expansion and formation of convoluted structures. After inspecting hundreds of electron micrographs, we were unable to identify KSHV capsids associated with these NR-derived convoluted membranes, suggesting that these membranes are not targets for primary envelopment.

To further advance understanding of KSHV nuclear egress mechanisms, we conducted live cell microscopy experiments to monitor capsid egress at the NR in real time. We employed fluorescently labeled KSHV genomes and capsids, and a fluorescent polar lipid dye, to monitor association of capsids with NR structures and subsequent trafficking events. After validating that fluorescent CLICK-labeled viral genomes co-localize with ORF65 small capsid protein in KSHV-capsid-sized puncta by immunofluorescence microscopy on fixed cells, we tracked their association with NR structures using the fluorescent polar lipid dye DiOC_6_ by spinning disc fluorescence microscopy. In parallel, we performed live-cell imaging of ORF65_mScarlet capsids in DiOC_6_-stained cells. These orthogonal approaches provide preliminary evidence that KSHV capsids associated with NR compartments can rapidly transit to the cytoplasm in minutes. While substantially more work is required to fully elucidate KSHV nuclear egress mechanisms at the NR, our study provides a roadmap towards development of more sensitive assays, combining approaches in labeling of viral genomes and capsids, as well as NR membranes. Future work should investigate the molecular machinery involved in remodeling these NR compartments, including whether the KSHV NEC is recruited to NR budding sites.

## MATERIALS AND METHODS

### Cell culture and reagents

All doxycycline-inducible iSLK-BAC16 cells were cultured in Dulbecco’s modified Eagle’s medium (DMEM) (Gibco) supplemented with 10% fetal bovine serum, 2 mM L-glutamine, 1% penicillin-streptomycin, and grown in 5% CO_2_ at 37°C. To maintain stability of episomal KSHV DNA in iSLK cells, parental cell cultures were maintained in 1 µg/mL puromycin (ThermoFisher) and 1 mg/mL hygromycin B (Invitrogen). iSLK-BAC16 cells were reactivated from latency with the addition of 1 mM sodium butyrate (Sigma) and 1 µg/mL doxycycline (Sigma).

### Transmission electron microscopy

iSLK-BAC16 cells were seeded in 10 cm dishes (VWR) at a density of approximately 1.5 × 10^5^ cells per dish (5% CO_2_ at 37°C). The next day, cells were reactivated from latency by the addition of 1 mM sodium butyrate and 1 µg/mL doxycycline and harvested at 24, 48, 72, or 96 h. Cells were harvested by trypsin digestion and centrifuged at 250 × *g* for 5 minutes. Samples were fixed for a minimum of 2 hours in 2.5% glutaraldehyde diluted in 0.1 M sodium cacodylate buffer. Following fixation, samples were rinsed three times for at least 10 minutes each with 0.1 M sodium cacodylate buffer. Secondary fixation was performed using 1% osmium tetroxide for 2 hours, followed by a brief rinse with distilled water. Samples were then placed in 0.25% uranyl acetate at 4°C overnight. Dehydration was carried out using a graded acetone series: 50% acetone for 10 minutes, 70% acetone for two 10 minute incubations, 95% acetone for two 10 minute incubations, and 100% acetone for two 10 minute incubations, followed by a final 10 minute incubation in dried 100% acetone. Samples were then infiltrated with Epon Araldite resin using a stepwise approach: 3:1 (dried 100% acetone to resin) for 3 hours, 1:3 (dried 100% acetone to resin) overnight, and finally 100% resin for two 3 hour incubations. Samples were embedded in 100% Epon Araldite resin and cured at 60°C for 48 hours. Ultrathin sections (~100 nm thick) were obtained using a Reichert–Jung Ultracut E ultramicrotome equipped with a diamond knife and were placed on 300-mesh copper grids. Sections were stained with 2% aqueous uranyl acetate for 10 minutes, followed by two 5 minute rinses with distilled water. Lead citrate staining was performed for 4 minutes, followed by a quick rinse with distilled water, and the grids were air-dried. Prepared samples were imaged using a JEOL JEM 1230 transmission electron microscope operating at 80 kV. Images were acquired using a Hamamatsu ORCA-HR digital camera. All TEM experiments were performed in three biological replicates.

### Immunofluorescence and image processing

Doxycycline-inducible iSLK-BAC16 cells were seeded at 2 × 10^5^ cells/mL on glass coverslips (Paul Marienfeld Gmb H & Co.; 0117580) in 12-well plates (VWR). The next day, cells were reactivated with 1 µg/mL doxycycline and 1 mM sodium butyrate and harvested at 72 h or 96 h. Coverslips were fixed in 4% paraformaldehyde for 15 min at room temperature, washed with PBS, and incubated in blocking/permeabilization (block/perm) buffer (Triton-X100 0.1% (vol/vol) (Sigma; 1002614889) and 1% human AB serum (Sigma) in PBS for 1 hour at RT. Coverslips were incubated with mouse anti-Lamin A/C (1:200, Sigma) and/or rabbit anti-CCTα (1:500) and/or rabbit anti-VAPA (1:200) antibodies overnight at 4°C in block/perm buffer. Experiments using iSLK-ORF65_mScarlet–ORF39_mNeon cells received rabbit polyclonal lamin B receptor (Proteintech Group Inc.; 12398-1-AP) diluted at 1:300. The next day, coverslips were washed three times with PBS followed by a 1 h incubation at room temperature with secondary donkey anti-rabbit AlexaFluor555 (Invitrogen; A31572) and chicken anti-mouse AlexaFluor647 (Invitrogen; A21463) for CCTα and LBR immunofluorescence experiments, and goat anti-mouse AlexaFluor555 (Invitrogen; A21422) and goat anti-rabbit AlexaFluor647 (Invitrogen; A21244) for VAPA immunofluorescence. All secondary antibodies were diluted 1:1,000 in block/perm buffer. After three additional PBS washes, coverslips were counterstained with Hoechst 33342 (1:5,000, 5 min, RT) and mounted on microscope slides (Fisherbrand; 12-550-15) using ProLong Gold Antifade (ThermoFisher, P36930).

### CLICK chemistry

iSLK-BAC16 cells were seeded at a density of 2 × 10⁵ cells per well onto either four-well chamber slides (Nunc Lab-Tek chambered cover glass; 155383) for live-cell imaging or glass coverslips for fixed-cell experiments and reactivated from latency as described above. For both conditions, cells were pulsed with 100  µM 5-vinyl-2′-deoxyuridine (VdU; Lumiprobe B2540) at 40  hours post-reactivation. For live-cell imaging, PINK was added at 44  hours post-reactivation to a final concentration of 100  µM and removed at 48  hours, followed by three washes with warm complete DMEM. Chamber wells were imaged 72 hours post-reactivation as described below. For fixed-cell imaging, coverslips were harvested at 72- and 96 hours post-reactivation, washed with PBS, and fixed in 4% paraformaldehyde as described above. Fixed cells were incubated overnight with a mouse monoclonal antibody against ORF65 (gift from S.-J. Gao) diluted 1:600 in blocking buffer. The following day, cells were incubated with chicken anti-mouse Alexa Fluor 647 secondary antibody (Invitrogen; A21463) for 1 hour, then washed three times with PBS. Coverslips were then inverted onto 40  µL droplets of 10  µM PINK in PBS within a humidified plastic container (moistened with a wet paper towel and sealed with Parafilm) and incubated for 4 hours at 37  °C with 5% CO₂. Coverslips were washed three times with PBS and incubated with DiOC₆ (final concentration 0.2  µL/mL in PBS) for 5 minutes at room temperature, followed by three PBS washes. Coverslips were then mounted onto microscope slides as described above. Live-cell CLICK chemistry experiments were successful using both PINK 1.0 and PINK 2.0 probes with comparable results.

### Spinning disc live-cell imaging

iSLK-BAC16 cells were seeded in four-well chamber slides (Nunc Lab-Tek; 155383) at a density of 2 × 10⁵ cells per well. The following day, cells were reactivated from latency with 1 µg/mL doxycycline and 1  mM sodium butyrate. CLICK chemistry experiments were pulsed with VdU and PINK as described above. At 72  hours post-reactivation, cells were stained with 10  µM BDP 630/650 lipid dye (Lumiprobe 1233) in DMEM without phenol red for 15  minutes, washed three times, and imaged in DMEM without phenol red, supplemented with 5% FBS. For dual-labeled virus experiments using iSLK-ORF65_mScarlet–ORF39_mNeon cells, reactivation and BDP 630/650 staining were performed as described above. Cells were imaged using a Zeiss Cell Observer spinning-disk confocal microscope with a 100 × oil immersion objective. Z-stacks ranged from 7 to 11 slices per stack, adjusted based on cell size and experiment duration. For extended imaging (>30  min), seven slices were used to minimize laser exposure and preserve cell health. Laser intensities were kept at the lowest settings to reduce phototoxicity. Imaging intervals varied depending on z-stack thickness, typically ranging from 40 seconds to 1.5 minutes (exact intervals noted in figure captions). Z-stacks were processed in Imaris for brightness and contrast enhancement, and individual timestamped images were exported using the Imaris capture tool for figures and videos.

### *De novo* infection and titering

iSLK-BAC16 cells were seeded in six-well plates (VWR) at a density of approximately 4 × 10^5^ cells per well (5% CO_2_ at 37°C). The next day, cells were reactivated from latency by the addition of 1 mM sodium butyrate and 1 µg/mL doxycycline and harvested at 24, 48, 72 h, 96 h, or 120 h. Virus-containing supernatant was harvested from iSLK-BAC16 cells at the indicated times by pelleting cellular debris at 3,300 × *g* for 5 minutes and then stored at −80°C until ready to titer the virus. To titer the virus, naïve 293 A cells were seeded at 150,000 cells/well in a 96-well plate. 5 µL of virus-containing supernatant was added to each well in technical duplicates. The plate was centrifuged at 800 × *g* for 2 h at 30°C. 24 h after infection, plates were fixed in 4%paraformaldehyde and counterstained with Hoechst 33342 (1:5,000, 5 min, RT) in block/perm buffer (see above). Infected cells were imaged using a Zeiss Axio Imager Z2 epifluorescence microscope equipped with a 20× objective. The microscope was calibrated to automatically image three randomly selected fields of view per well. Image processing and quantification were performed using Imaris software. Masks were generated for the Hoechst (nuclei) and GFP (infected cells) channels, and the number of nuclei overlapping with GFP-positive signals was quantified automatically. All images were reviewed manually to exclude artifacts such as monolayer tears or debris. For each biological replicate (*n* = 3), the average number of cells showing overlap between green and blue signals was calculated from technical duplicates.

### Quantitative reverse-transcription PCR

iSLK-BAC16 cells were seeded in six-well plates (VWR) at a density of approximately 400,000 cells per well (5% CO_2_ at 37°C). The next day, cells were reactivated from latency by the addition of 1 mM sodium butyrate and 1 µg/mL doxycycline and harvested at 24, 48, or 72 h. RNA was isolated from cells with the RNeasy Plus Kit (Qiagen), and 500 ng total RNA was reverse transcribed with the Maxima H Minus First Strand cDNA Synthesis Kit (Thermo Cat: K1652). For optimal first-strand synthesis, primer volume was made up from half Oligo(dT)18 and half Random hexamer primers. A CFX96 Touch Real-Time PCR Detection System (Bio-Rad) and Luna Universal qPCR Master Mix (NEB cat: M3003L) were used to perform Real-Time PCR. Changes in mRNA levels were calculated by the ΔCt method and normalized using 18S rRNA as a reference gene. Four biological replicates were analyzed, each processed as two technical replicates. The following primer sets were used in this assay:

*18S* F: 5′-TTCGAACGTCTGCCCTATCAA-3′; R: 5′-GATGTGGTAGCCGTTTCTCAGG-3′ *nRTA* F: 5′-TCCAGTTTTGCTCCCCACTG -3′; R: 5′- TTCTGCCGTATTGTAGGCGG-3′

*ORF57:* 5′-TCCAGTTTTGCTCCCCACTG-3′ R: 5′-TTCTGCCGTATTGTAGGCGG-3′

*SOX:* 5′-ACCACGGAGTCTGACGTCTA-3′ R: 5′-ACGATCGAACTCTGCAGCAA-3′ *vGPCR:* 5′-GTACTGACATCCGCTGCACT-3′ R: 5′-TCATGTTTCCCGCGTTCTCA-3′

*K2:* F: 5′-TCTCTTGCTGGTCCGGTTCAC-3′ R: 5′-CGGTACGGTTAACAGAGGTCG-3′

*ORF65:* F: 5′-TGGCTCGCATGAATACCCTG-3′ R: 5′-CTGCAGATGATCCCCGCTTT-3′

### Nuclear-associated episome quantification

iSLK-BAC16 cells were seeded in six-well plates (VWR) at a density of approximately 400,000 cells per well (5% CO_2_ at 37°C). The next day, cells were reactivated from latency by the addition of 1 mM sodium butyrate and 1 µg/mL doxycycline and harvested at 24 h, 48 h, 72 h, 96 h, or 120 h. DNA was harvested from cells using 200 µL/well of virus lysis buffer (20 mM Tris-HCl, pH 7.4, 300 mM NaCl, and 2.5% NP-40). Samples were sonicated 10 seconds to break nuclear membranes, diluted with 200 µL of dH_2_O to create a stock, and 25 µL of that stock was further diluted with 975 µL of dH_2_O to create a working stock for use in qPCR and stored at −80°C. Prior to qPCR, samples were spun down at 3,300 × *g* for 5 minutes, and only the top portion of the sample was used. qPCR (or qPCR) was carried out as described above with primers specific to KSHV ORF26 and β-actin. Changes in KSHV genome copy number were calculated by the ∆∆Ct method relative to latent cells and normalized to β-actin. Three biological replicates were analyzed, each processed as two technical replicates. The following primer sets were used in this assay:

*ORF26:* 5′-CAGTTGAGCGTCCCAGATGA-3′R: 5′-GGAATACCAACAGGAGGCCG-3′

*β-actin:* 5′-CTTCCAGCAGATGTGTGATCA-3′R: 5′-AAAGCCATGCCAATCTCATC-3′

### Graphing and statistical analysis

GraphPad Prism Version 10.4.1 was used to generate graphs and complete statistical analysis. One-way ANOVA comparing means between groups (i.e., latent vs. 24 h, latent vs. 48 h, etc.) with a Tukey multiple comparison test was used to compare differences in gene expression between groups. *P*-values < 0.05 were considered significant and denoted as the following: <0.05 (*), <0.01 (**), <0.001 (***), <0.0001 (****), and > 0.05 was denoted as not significant (ns).

### Imaging and data processing

Z-stacks were acquired using a Zeiss LSM 880 fluorescence microscope (100× oil objective) and processed into maximum intensity projections using Zeiss Black software. Images were further analyzed in FIJI (ImageJ), with a global contrast increase of 0.34% for VAPA experiments. Notably, 96 h samples exhibited intense CCTα staining, suggesting upregulation or aggregation. To compensate, the A568 laser power was reduced from 4 to ~ 2.6 when imaging 96 h samples. Figure 3A depicts the diversity of NI observed in KSHV-infected cells. Each cartoon is paired with a representative TEM image from the published data set to convey a clear understanding of the appearance of these structures; these include a first-order NI from Fig. 5, a second-order NI from Fig. 3B, a third-order NI from Fig. 6, and a convoluted membrane structure from Fig. 5. The legend associated with Fig. 5 is designed to help the reader inspect TEM images and identify key structures in the raw images and marked-up images, including envelope, genome, capsid, ONM, INM, lamina, and absence of lamina. The images associated with the legend were reproduced from our primary image data set and include a capsid presented in Fig. 8.

## Data Availability

Additional electron micrographs for this study are available via the Dryad open data publishing platform at https://doi.org/10.5061/dryad.qbkh18v9.

## SUPPLEMENTAL MATERIAL

Movie S1 jvi.00588-25-s0001.mov Tour of CLICK labeled genomes shown in Fig. 9D associating with and exiting the DiOC6 labeled NR. Cells were subject to live-cell imaging as described for Fig. 9. Movie cycles through time stamps over the course of egress, and the cell is rotated to appreciate 3D architecture.

Movie S2 jvi.00588-25-s0002.mov Zoomed-in view of CLICK labeled genomes shown in Fig. 9D and Movie S1.

Movie S3 jvi.00588-25-s0003.mov Tour of ORF65-mScarlet capsids shown in Fig. 10C associating with and exiting the DiOC6 labeled NR. Cells were subject to live-cell imaging as described in Fig. 10. Movie cycles through time stamps over the course of egress.

Movie S4 jvi.00588-25-s0004.mov Zoomed-in view of a specific event shown in Fig. 10C and Movie S3 where three ORF65-mScarlet capsids are seen within the NR, moving to the cytoplasmic face of the NR, and exiting to the cytosol.

ASM does not own the copyrights to Supplemental Material that may be linked to, or accessed through, an article. The authors have granted ASM a non-exclusive, world-wide license to publish the Supplemental Material files. Please contact the corresponding author directly for reuse.
